# Difficult Airway Prediction in Infants with Apparently Normal Face and Neck Features

**DOI:** 10.3390/jcm13154294

**Published:** 2024-07-23

**Authors:** Ivana Petrov, Zorana Stankovic, Ivan Soldatovic, Ana Tomic, Dusica Simic, Miodrag Milenovic, Vladimir Milovanovic, Dejan Nikolic, Nevena Jovicic

**Affiliations:** 1 University Children’s Hospital, Tirsova 10, 11000 Belgrade, Serbia; zorana0508@gmail.com (Z.S.); dusicasimic2@gmail.com (D.S.); vmilovanovic1972@yahoo.com (V.M.); denikol27@gmail.com (D.N.); jovicic.nevena@gmail.com (N.J.); 2 Faculty of Medicine, University of Belgrade, 11000 Belgrade, Serbia; soldatovic.ivan@gmail.com (I.S.); milenx67@gmail.com (M.M.); 3 University Clinical Centre of Serbia, Pasterova 2, 11000 Belgrade, Serbia; anatomic9977@gmail.com

**Keywords:** infants, airway assessment, difficult airway predictors, difficult facemask ventilation, difficult intubation

## Abstract

**Background/Objectives**: Prediction of a difficult airway during pre-anesthetic evaluation is of great importance because it enables an adequate anesthetic approach and airway management. As there is a scarcity of prospective studies evaluating the role of anthropometric measures of the face and neck in predicting difficult airways in infants with an apparently normal airway, we aimed to identify the aforementioned predictors of difficult facemask ventilation and intubation in infants. **Methods**: A prospective, observational study that included 97 infants requiring general endotracheal anesthesia was conducted. Anthropometric and specific facial measurements were obtained before ventilation and intubation. **Results**: The incidence of difficult facemask ventilation was 15.5% and 38.1% for difficult intubation. SMD (sternomental distance), TMA (tragus-to-mouth angle distance), NL (neck length) and mouth opening were significantly lower in the difficult facemask ventilation group. HMDn (hyomental distance in neutral head position), HMDe (hyomental distance in neck extension), TMD (thyromental distance), SMD, mandibular development and mouth opening were significantly different in the intubation difficulty group compared to the non-difficult group. HMDn and HMDe showed significantly greater specificities for difficult intubation (83.8% and 76.7%, respectively), while higher sensitivities were observed in TMD, SMD and RHSMD (ratio of height to SMD) (89.2%, 75.7%, and 70.3%, respectively). Regarding difficult facemask ventilation, TMA showed greater sensitivity (86.7%) and SMD showed greater specificity (80%) compared to other anthropometric parameters. In a multivariate model, BMI (body mass index), COPUR (Colorado Pediatric Airway Score), BOV (best oropharyngeal view) and TMA were found to be independent predictors of difficult intubation, while BMI, ASA (The American Society Physical Status Classification System), CL (Cormack–Lehane Score), TMA and SMD predicted difficult facemask ventilation. **Conclusions**: Preoperative airway assessment is of great importance for ventilation and intubation. Patient’s overall condition and facial measurements can be used as predictors of difficult intubation and ventilation.

## 1. Introduction

Preoperative airway assessment, based on history and physical findings, is intended to detect potential difficulties with establishing and maintaining oxygenation. Physical examination may include assessment of facial features, anatomical measurements and landmarks [1]. Pre-anesthetic identification of difficult airways is crucial as it informs the plan for anesthetic care and prompts early mobilization of additional resources [2]. Failing to assess the airway correctly by anesthesiologists results in poor management [3].

Facing a difficult airway can lead to severe hypoxemia and adverse events, and these issues are amplified in infants. Large occiput, more anterior larynx, larger tongue relative to infant’s oral cavity and large, omega-shaped epiglottis can make direct laryngoscopy (DL) in infants much harder than in older children [4]. Furthermore, greater oxygen consumption, smaller functional residual capacity and small closing capacity are the reasons why they desaturate very fast, which gives less time to intervene before failure to oxygenate and ventilate have serious consequences, including post-anoxic coma and death [5]. These unique anatomical and developmental characteristics as well as the physiology of infants are the main reasons why most screening tests for preoperative airway assessment and scoring systems cannot be extrapolated from the adult population.

To date, there are no specific predictive tests for difficult airways in infants, especially those with apparently normal craniofacial proportions [6]. Airway assessment tests measuring anthropometric parameters of the face and neck have limited clinical value because of low sensitivity, moderate specificity and high variability [7]. The widely used modified Mallampati test is not feasible in infants since it requires a cooperative patient; however, BOV (best oropharyngeal view) can be assessed without tongue protrusion [8].

The aim of our study was to identify predictors of difficult facemask ventilation and intubation in infants without obvious dysmorphic features of the face and neck undergoing elective surgical procedures under general endotracheal anesthesia.

## 2. Materials and Methods

### 2.1. Subjects

Infants requiring endotracheal intubation for elective surgery were enrolled after obtaining written informed consent from their parents or legal guardians. A sample size of 60 and 37 infants (with easy and difficult intubation, respectively), with an effect size of at least 0.6 had 80% power to detect significant differences between examined groups based on anthropometric measures. Similarly, a sample size of 82 and 15 infants (with easy and difficult facemask ventilation) with an effect size of at least 0.8 had 80% power to detect significant differences in the anthropometric measures. Study cases were selected using inclusion and exclusion criteria in consecutive order.

The exclusion criteria were congenital and acquired craniofacial malformations, and syndromes and conditions associated with anticipated difficult airway management, such as cervical spine deformities and cardiac and maxillofacial surgery patients. Some of these conditions could precipitate pain during manipulation of the head and neck, so we were sure that we were not causing any major discomfort during measurements. 

### 2.2. Study Design

This prospective observational study was conducted at the University Children’s Hospital in Belgrade from January 2022 to January 2023.

### 2.3. Data Curation

Pre-operative assessment was done by an anesthesiologist the day before surgery and included collecting demographic data, anamnesis, calculating BMI (body mass index) and defining the ASA score (The American Society of Anesthesiology Physical Status Classification System) [9]. Infants with BMIs between the 85th and 95th percentiles were considered overweight, over the 95th percentile obese and under the 3rd percentile underweight based on age and gender-specific charts from the World Health Organization. Measurements were taken by an anesthesiologist with the help of a nurse, requiring only head extension for some of them, which was done without major challenges and discomfort to the infants. Anthropometric face and neck measurements taken with a measurement tape were: hyomental distance in neutral head position (HMDn, obtained as distance in mm from hyoid to the lower end of the chin) and in neck extension (HMDe), hyoid-thyroid distance (HT, from the hyoid bone to the thyroid notch), neck circumference (NC, measured with superior border of the measuring tape placed just below the laryngeal prominence), neck length (NL, obtained as the distance from upper margin of the hyoid bone to the jugular notch), neck mobility (measured in degrees, in neck extension and flexion), lower-lip-to-chin distance (LCD, from the lower end of the lower lip to the lower end of the chin), tragus-to-mouth angle (TMA), thyromental distance (TMD, from thyroid notch to the tip of the chin, with the head fully extended), sternomental distance (SMD, from the upper border of manubrium sterni to the mentum) and mouth opening. Maximal mouth opening was measured with a ruler as the distance between gingival margins, if the child was toothless, or upper and lower frontal incisors, by placing a thumb and forefinger in the infant’s mouth with slight pressure. The measurement was performed as an attempt and since it is not painful, the infants did not resist the procedure. The ratio of height to TMD (RHTMD) as well as the ratio of height to SMD (RHSMD) were then calculated [6,8].

Assessment of oropharyngeal structures was done by determining the best oropharyngeal view (BOV), proposed by Aggarval et al. [8], with the child’s mouth open, but without protrusion of the tongue, unlike the modified Mallampati test [10]. BOV was classified as follows: Grade 0—soft palate, fauces, uvula, pillars visualized; Grade 1—soft palate, fauces, uvula visualized; Grade 2—soft palate, base of uvula; Grade 3—soft palate not seen [8].

The Colorado Pediatric Airway Score (COPUR) was calculated by rating chin size, interdental opening, previous intubation or obstructive sleep apnea, visualization of uvula and range of motion of the neck on the four-point scale. COPUR score > 10 predicted difficult intubation [11].

Airway assessment in the operating room was done by a senior anesthesiologist blinded to the study by grading facemask ventilation (GMV) according to the American Society of Anesthesiologists Practice Guidelines for Management of the Difficult Airway, as easy or difficult requiring multiple manipulations, such as the use of oropharyngeal tube, two hands, facemask adjustments or adjustments of the head and neck position [1]. In the case of difficult mask ventilation and intubation, Pediatric Difficult Airway Guidelines issued by the American Society of Anesthesiologists were followed [1].

Following direct laryngoscopy, the laryngoscopic view was classified using the Cormack–Lehane score (CL) as Grade 1—most of the cords visible; Grade 2a—posterior cord visible; 2b—only arytenoids visible; Grade 3a—epiglottis visible and liftable; 3b—epiglottis adherent to the pharynx and Grade 4—no laryngeal structures seen [12,13]. CL scores of 3 and above were considered difficult laryngoscopy.

A quantitative scale of intubation difficulty—intubation difficulty score (IDS)—was used for the evaluation of endotracheal intubation [14]. IDS includes 7 variables that increase the degree of difficulty from 0 (predefined as ideal intubation) by one point for each of the following: supplementary attempts, different operators, alternative techniques used, CL grade, lifting force required, laryngeal pressure applied and position of vocal cords (abduction adduction). IDS > 5 was considered as difficult intubation. This precise way of assessing difficult intubation eliminated interobserver variations, although the procedure was done by an experienced anesthesiologist in all the cases.

Infants were premedicated with midazolam 0.5 mg/kg administered orally 30 min before intervention or 0.1 mg/kg via iv if the iv line was placed. Anesthesia induction was intravenous: thiopentone 5 mg/kg or propofol 2.5 mg/kg, fentanyl 3 mcg/kg and non-depolarizing muscle relaxant rocuronium 1 mg/kg or cis-atracurium 0.15 mg/kg. Facemask ventilation difficulty was quantified after induction. Anesthesia maintenance was intravenous or inhalatory, depending on the anesthesiologists’ decision. Patients were intubated with a Macintosh-type blade in the supine position, without additional elevation of the occipital part of the head.

### 2.4. Ethical Considerations

This study was conducted in accordance with the Declaration of Helsinki and approved by the Institutional Review Board of the University Children’s Hospital in Belgrade (decision No. 14/87) and Faculty of Medicine, University of Belgrade (decision No. 17/I-11).

### 2.5. Statistical Analysis

Results are presented as count (%) and mean ± standard deviation. Groups are compared using parametric (*t*-test) or non-parametric tests (Chi-squared or Fisher’s Exact test, as appropriate). Binary univariable and multivariable logistic regressions were performed to assess correlations between facemask ventilation and intubation difficulties as outcomes and clinical characteristics as predictors. Sensitivity and specificity were calculated to assess the association between the outcome and exposure variables. All p-values less than 0.05 were considered significant. All data were analyzed using SPSS 29.0 (IBM Corp. Released 2023. IBM SPSS Statistics for Windows, Version 29.0. Armonk, NY, USA: IBM Corp.).

## 3. Results

### 3.1. Demographics

Our study included a total of ninety-seven infants. Distribution of both genders was nearly equal, with males accounting for 49.5% and females for 50.5% of the sample. The average age of patients was 4.6 months, with a mean length of 62 cm and an average weight of 5.9 kg. The majority of infants were within the normal BMI range (68%), with the underweight category being the second most prevalent (22.7%).

Overall, 49.5% of the patients had an ASA 1 score, 83.1% had a desirable COPUR Score below 10 and 35.1% had a BOV score of 1, while 34% of infants had the most unfavorable BOV score of 3. The majority of infants exhibited normal jaw development (73.2%) and mouth opening width between 20 and 40 mm (67.4%). Additionally, a substantial portion of participants (39.2%) demonstrated a Cormack–Lehane (CL) Grade of 2a, 29.9% had a CL Grade of 2b, while a minority of patients (8.2%) presented with a CL Grade of 3a.

### 3.2. Difficult Facemask Ventilation

Table 1 shows that 15 infants (15.5%) had difficulty with facemask ventilation. There were no apparent distinctions in demographics, ASA physical status as well as intubation difficulty between the two groups. There was a substantial difference between the two groups based on BMI, COPUR, BOV and CL scores.

Table 2 displays significantly lower TMA, SMD and NL measures in the difficult ventilation group, along with significant discrepancies in mouth opening range. No discrepancies were found between the groups examined based on the other variables examined in the study.

The identified parameters that showed statistical significance were further analyzed using binary logistic regression analysis. According to the multivariate logistic regression analysis, BMI, COPUR score, BOV and TMA values were identified as independent predictors of difficult ventilation, as shown in Table 3.

### 3.3. Difficult Intubation

Based on the data provided in Table 4, it was observed that a total of 37 (38.1%) infants experienced difficult tracheal intubation. Statistically significant differences were found in BMI, ASA score, preoperative airway assessment scores (COPUR and BOV) and CL grade based on intubation difficulty. Infants who experienced difficult intubation were found to have a significantly more common physical status of ASA score of 3, a COPUR score above 10, BOV scores of 2 and 3 and CL scores of 2b and 3a.

The results of correlation analyses indicated a positive, medium-strength correlation between BOV values and CL Scores (r = 0.514, *p* < 0.001) and IDS (r = 0.373, *p* < 0.001).

The analysis of jaw and neck anthropometric parameters (Table 5) revealed that HMDn, HMDe, TMD and SMD measurements were significantly reduced in the difficult intubation group. No differences were noticed between the examined groups in terms of other measured anthropometric parameters, including neck motility range. Underdeveloped and hypoplastic jaws along with a mouth opening range of less than 40 mm were significantly more common in the difficult intubation group.

Additional multivariate regression analyses revealed that five factors—BMI (percentile), ASA score, greater CL score, TMA and SMD—were identified as significant independent predictors of difficult intubation (Table 6).

Table 7 displays the results of the ROC analysis. Our study revealed that HMDn, HMDe, TMD, SMD and RHSMD are significant classificatory markers, and TMD and SMD exhibit notably greater sensitivity rates of approximately 0.9 and 0.8, respectively, to classify patients for difficult intubation. On the other hand, HMDn, HMDe and LCD have better specificities. Other test results did not demonstrate statistical significance for difficult intubation. According to our research, TMA and SMD are two statistically significant predictive values for difficult ventilation, both exhibiting a sensitivity of approximately 0.8.

## 4. Discussion

### 4.1. Difficult Facemask Ventilation

Our study showed the incidence of difficult facemask ventilation in infants to be 15.5%, which is higher than in previous studies in children. This was presumably because of the young age of our subjects since the incidence of facemask ventilation in infants has rarely been assessed in the available literature. According to the literature, age is independently associated with DMV [15].

Valois Gomez et al. reported an incidence of 6.6% for unexpected and 0.5% for expected DMV among otherwise healthy children from 0 to 8 years old, while the incidence of difficult intubation (DI) was 1.2%, and no association was found between DBMV and DI [15]. On the contrary, other studies showed that the incidence of DMV increases when tracheal intubation is difficult, with an incidence from 7.6% to 13% [16,17]. Our study showed that difficult intubation occurred twice as often in the difficult ventilation group, but it was not statistically significant based on further analysis. Garcia-Marcinkiewicz et al.’s analysis of a multicenter registry of children with difficult tracheal intubation found an incidence of difficult mask ventilation of 9%, which was more likely in infants, patients with increased weight, being < 5th percentile in weight for age, glossoptosis and limited mouth opening [18]. These authors included children with an airway-related diagnosis or syndrome from the Pediatric Difficult Intubation (PeDI) registry, who were excluded from our study. Similarly, mouth opening width was found to be an important factor in assessing the difficulty of ventilation in our study. It was observed that patients who experienced difficult ventilation typically exhibited a mouth opening range of 20–40mm.

A significant proportion of overweight and all obese infants in our study had facemask ventilation difficulties, which is similar to data from a study that reported the incidence of DMV to be 2.1% in nonobese children and 8.7% in obese children [19]; however, another study found that BMI did not have a significant effect on the difficulty of mask ventilation [15].

Preoperative air assessment of our patients, by determining COPUR and BOV scores as well as CL scores during direct laryngoscopy, revealed that the majority of infants who experienced difficult ventilation had COPUR scores above 10, a BOV score of 3 and a CL score of 2b and higher.

After anthropometric measurements of the face and neck were conducted, significant reductions in measurements of TMA, SMD and NL were observed within the difficult ventilation cohort. Results from the ROC analysis revealed cut-off values for TMA of 6.4 cm (sensitivity, 0.867; specificity, 0.488) and 6.3 cm for SMD (sensitivity, 0.8; specificity, 0.55) for predicting difficult facemask ventilation. Based on the recorded findings, tragus-to-mouth angle distance (TMA) and BMI were the only reduced anthropometric parameters defined as independent predictors of DMV in infants. There was no significant reduction in parameters defining the chin, implicating the importance of midface hypoplasia as a cause of difficult facemask ventilation. Further analysis showed that along with lower TMA measurements and higher BMI, higher BOV and COPUR scores were independent predictors of difficult facemask ventilation. We lack comparative studies in infants that we can reference against our findings.

### 4.2. Difficult Intubation

This study showed a difficult intubation incidence of 38.1%, defined as an IDS score > 5, which is consistent with the current evidence showing that younger children have a higher risk of difficult intubation [17]. According to the literature, the incidence of difficult intubation (DI) in children varies from 0.06% to 3% and is found to be highest in children under the age of one and children who are underweight, have an ASA physical status of 3 or 4 or have a Mallampati score of 3 or 4 [17,20,21].

A recent APRICOT study reported difficult intubation in 0.28% of patients, with higher incidences in neonates and infants (1% and 1.1%, respectively) [22]. Analysis of difficult tracheal intubation—defined as two failed attempts of direct laryngoscopy in neonates and infants—in the European multicenter observational study (Neonate and Children audit of anesthesia practice in Europe: NECTARINE), showed an incidence of 5,8% and it was unexpected in two-thirds of the cases [16].

We believe that the generally accepted belief that “the incidence of unexpected difficult airway in children is low”, is questionable. The evaluation of facemask ventilation as well as direct laryngoscopy and intubation in our study was done by a senior specialist to avoid association of the training level of the airway provider with the first-attempt intubation success.

Our study results showed that both underweight and obese infants were more likely to experience DI. Most of the studies refer to low BMI as a predictor of difficult laryngoscopy [20,23]. Additionally, a higher ASA score (3 and 4) was associated with a significantly increased incidence of DI, similar to earlier findings by Heinrich et al., which showed that underlying diseases might also contribute to the risk [20].

Subtle signs of mandibular hypoplasia can be easily missed in neonates and infants. However, what is important is to take face and neck proportions into consideration. There are potential limitations to using anthropometric measurements in clinical practice, considering variability in infants’ growth and development. The mandible is not developed in infants and grows later in life; thus, we believe that excluding newborns and older children made our group of patients, from 29 days to 12 months old, comparable. Observing the child’s face from the front as well as from the lateral profile is important for the detection of mandibular hypoplasia, which was identified in 26.8% of infants in our study. This group of patients exhibited more frequent instances of difficult intubation, along with infants who had a mouth opening of less than 20 mm. According to the literature, interincisor distance correlates with Cormack–Lehane Grade [24]. Ahmadi et al. provided normative data on the association between age and maximal mouth opening (MMO) in healthy infants under 12 months of age; the mean MMO was 36.7 mm (95% CI: 34.8–38.6) [25].

Although most of the infants had a BOV score of 1, we recorded a high percentage of BOV scores of 2 and 3 among our patients, meaning that visibility of oropharyngeal structures was not optimal very often and only soft palate and uvula were seen during the preoperative examination. There was a correlation between preoperatively assigned BOV grades and DI, as children with DI tended to have BOV grades 2 and 3. It appeared that BOV is an important predictor of DI, but additional multivariate regression analysis did not identify this score as an independent predictor. Manabe et al. described the same test, by the name of NT-MMT (no tongue protrusion Mallampati classification) that was more accurate in predicting difficult tracheal intubation than mMMT in adults, possibly because tongue protrusion may sometimes hide the oropharynx when the distance of the mouth opening is short, which leads to false positive estimations of difficulty [26].

To date, there is little data on anthropometric measures that can predict difficult airway in pediatric patients, especially in infants. HMD is used for the estimation of mandibular space and was identified as a predictor of difficult airway status in previous studies [27]. The findings of our study revealed that HMDn values of less than 3.05 cm (with a sensitivity of 51.7% and a specificity of 83.8%) and HMDe values less than 4.05 cm (with a sensitivity of 45.9% and a specificity of 76.7%) were the cut-off values that forecast DI.

TMD < 4.7 cm (sensitivity, 89.2%; specificity, 33.3%) and SMD < 6.5 cm (sensitivity, 75.7%; specificity, 60%) were determined as cut-off values for DI. Accordingly, TMD, SMD and RHSMD are anthropometric parameters with higher sensitivities hence they can be helpful in the identification of infants in whom intubation will be truly difficult.

Rafique et al. recorded that a median TMD of 3.5 cm was significantly low in Cormack–Lehane Grade 3 compared to Grades 1 and 2 among children less than 5 years of age [28]. On the other hand, Inal et al. [29] determined the cut-off value for TMD as 5.5 cm in children 5–11 years old, while Wang et al. reported TMD of 4.1–5.8 cm in Chinese children aged 4–12 years [30].

In our multivariable regression analysis, TMA, SMD, BMI, ASA and CL were defined as significant predictors of DI. The angle of the mandible is not developed in children, so measuring the distance from ear tragus-to-mouth angle (TMA) or tragus-to-nares (Tn) can be a measure of horizontal mandibular length, which correlates with CL grade in children younger than 5 years [28].

Our study did not find a correlation between difficult intubation and RHTMD. Similarly, Sumer et al. observed that the diagnostic accuracy of RHTMD was lower among other tests in children aged 5–12 years [2], while Ray et al. found RHTMD to be a better predictor of restricted laryngoscopy view than RHSMD in children aged 1–12 years [31]. Maddali et al. demonstrated in their study that there is a good correlation between RHTMD with a cut-off value of 15.77 cm with difficult laryngoscopy views in pediatric patients below 5 years of age [23]. According to our results, RHSMD appeared as a significant marker for difficult intubation, with a greater sensitivity rate, but again, data from the literature considered children older than one year.

There was no association between neck circumference and difficult intubation, similar to what Nafiu et al., Mansano et al. and Kilic et al. observed in their studies [24,27,32]. On the other hand, Maddali et al. reported that NC ≥ 21.4 cm was associated with a poor laryngoscopic view in children below 5 years [23]. According to our study, neck length was significantly lower in the difficult facemask ventilation group.

This research was limited by a comparatively modest sample size, perhaps limiting the applicability of the results to a wider cohort of infants. Additionally, it should be noted that this study was carried out exclusively at a single institution, thereby constraining the diversity of the study sample. This work offers interesting insights into the association between facial and neck characteristics of infants and the difficulties faced during facemask ventilation and intubation. However, it is important to acknowledge the limitations of this study, which underscore the necessity for additional research in this area.

## 5. Conclusions

Since taking anthropometric measurements of the face and neck is time-consuming, we would like to emphasize the importance of identifying independent predictors, which are also the most reliable and feasible for clinical practice. The present study reports high BMI, BOV and COPUR scores and lower TMA as independent predictors of difficult facemask ventilation, and high BMI, ASA and CL scores, as well as lower TMA and SMD as independent predictors for difficult intubation in infants. We conclude that, since the Mallampati test is not applicable in small children, BOV can be helpful in the preoperative identification of difficult airways in infants.

## Figures and Tables

**Table 1:** Patient demographics and scores based on mask ventilation difficulty.

	Easy Ventilation (*n* = 82)	Difficult Ventilation (*n* = 15)	*p* Value
Age (months)	4.78 ± 3.42	3.70 ± 3.43	0.263 ^a^
Weight (in kilograms)	5.9 ± 1.9	5.9 ± 2.5	0.891 ^a^
Body length (in centimeters)	62.1 ± 10.9	60.1 ± 8.2	0.505 ^a^
Sex			
Male	40 (83.3%)	8 (16.7%)	0.746 ^b^
Female	42 (85.7%)	7 (14.3%)	
BMI			
Underweight	18 (81.8%)	4 (18.2%)	<0.001 ^d^
Normal	62 (93.9%)	4 (6.1%)	
Overweight	2 (33.3%)	4 (66.7%)	
Obese	0	3 (100%)	
BMI percentile	33.2 ± 28.3	52.7 ± 39.7	0.086 ^a^
ASA score			
1	41 (85.4%)	7 (14.6%)	0.237 ^b^
2	32 (88.9%)	4 (11.1%)	
3	9 (69.2%)	4 (30.8%)	
COPUR Score			
10 or less	72 (88.9%)	9 (11.1%)	0.016 ^c^
Above 10	10 (62.5%)	6 (37.5%)	
Best Oropharyngeal View (BOV)			
0	3 (100%)	0	0.001 ^d^
1	32 (94.1%)	2 (5.9%)	
2	25 (92.6%)	2 (7.4%)	
3	22 (66.7%)	11 (33.3%)	
CL Score			
1	22 (100%)	0	0.011 ^d^
2a	33 (86.8%)	5 (13.2%)	
2b	21 (72.4%)	8 (27.2%)	
3a	6 (75%)	2 (25.0%)	
Difficult intubation			
Yes	29 (78.4%)	8 (21.6%)	0.188 ^b^
No	53 (88.3%)	7 (11.7%)	

Results are presented as count (%) or mean ± sd; ^a^ *t*-test; ^b^ Pearson Chi-squared test; ^c^ Fisher’s Exact test; ^d^ Chi-squared test for trends.

**Table 2:** Anthropometric parameters of the jaw and neck based on mask ventilation difficulty.

	Easy Ventilation (*n* = 82)	Difficult Ventilation (*n* = 15)	*p* Value
Anthropometric measurements of the jaw and neck (mm)			
HMDn	31.1 ± 6.7	31.1 ± 5.8	0.989 ^a^
HMDe	43.6 ± 7.9	44 ± 6.6	0.830 ^a^
LCD	22.4 ± 2.9	22.7 ± 2.8	0.672 ^a^
TMA	63.7 ± 7.9	58.8 ± 6.7	0.026 ^a^
TMD	42.1 ± 9.6	38.4 ± 8.2	0.175 ^a^
RHTMD	15.3 ± 3.6	16.1 ± 3.4	0.437 ^a^
SMD	64.3 ± 10.5	57.7 ± 7.8	0.023 ^a^
RHSMD	9.8 ± 1.9	10.6 ± 2.1	0.157 ^a^
NC	233.8 ± 25.9	225 ± 36.1	0.382 ^a^
NL	54.2 ± 13.8	46.6 ± 8.4	0.007 ^a^
Mandibular development			
Normal jaw	62 (87.3%)	9 (12.7%)	0.219 ^b^
Small, hypoplastic jaw	20 (76.9%)	6 (23.1%)	
Mouth opening width (mm)			
>40	30 (96.8%)	1 (3.2%)	0.032 ^c^
≤40	52 (78.8%)	14 (21.2%)	
Neck motility (in degrees)			
Over 120	74 (87.1%)	11 (12.9%)	0.087 ^c^
60–120	8 (66.7%)	4 (33.3%)	

Results are presented as count (%) or mean ± sd; ^a^ *t*-test; ^b^ Pearson Chi-squared test; ^c^ Fisher’s Exact test.

**Table 3:** Ventilation difficulty models.

	Univariable		Multivariable Forward Method	
	OR (95% CI)	*p* Value	OR (95% CI)	*p* Value
BMI percentiles	1.020 (1.002–1.039	0.029	1.051 (1.019–1.085)	0.002
COPUR	4.800 (1.408–16.366)	0.012	7.599 (1.405–41.088)	0.019
BOV	3.368 (1.469–7.724)	0.004	3.524 (1.237–10.041)	0.018
CL Score	2.382 (1.215–4.673)	0.012		
TMA	0.917 (0.848–0.992)	0.031	0.843 (0.729–0.975)	0.022
TMD	0.953 (0.888–1.022)	0.179	1.092 (0.983–1.213)	0.101
SMD	0.933 (0.878–0.992)	0.027		
RHSMD	1.213 (0.926–1.590)	0.160		
NL	0.941 (0.884–1.001)	0.054		
Mouth opening ≤ 40	8.077 (1.011–64.517)	0.049		
Neck motility 60–120	3.364 (0.866–13.067)	0.080		

**Table 4:** Anthropometric parameters of the jaw and neck based on intubation difficulty.

	Easy Intubation (*n* = 60)	Difficult Intubation (*n* = 37)	*p* Value
Age (months)	4.46 ± 3.19	4.85 ± 3.81	0.594 ^a^
Weight (in kilograms)	6.1 ± 1.8	5.6 ± 2.3	0.232 ^a^
Body length (in centimeters)	62.2 ± 10.5	61.2 ± 10.7	0.667 ^a^
Sex			
Male	29 (60.4%)	19 (39.6%)	0.773 ^b^
Female	31 (63.3%)	18 (36.7%)	
BMI			
Underweight	8 (36.4%)	14 (63.6%)	0.007 ^c^
Normal	48 (72.7%)	18 (27.3%)	
Overweight	3 (50%)	3 (50%)	
Obese	1 (33.3%)	2 (66.7%)	
BMI percentile	41.7 ± 30.1	27.3 ± 30.4	0.084 ^a^
ASA score			
1	37 (77.1%)	11 (22.9%)	<0.001 ^d^
2	22 (61.1%)	14 (38.9%)	
3	1 (7.7%)	12 (92.3%)	
Previous intubation			
Easy	14 (56%)	11 (44%)	0.067 ^c^
Difficult	0	3 (100%)	
COPUR Score			
10 or less	60 (74.1%)	21 (25.9%)	<0.001 ^b^
Above 10	0	16 (100%)	
Best Oropharyngeal View (BOV)			
0	3 (100%)	0	<0.001 ^d^
1	29 (85.3%)	5 (14.7%)	
2	12 (44.4%)	15 (55.6%)	
3	16 (48.5%)	17 (51.5%)	
CL Score			
1	20 (90.9%)	2 (9.1%)	<0.001 ^d^
2a	28 (73.7%)	10 (26.3%)	
2b	12 (41.4%)	17 (58.6%)	
3a	0	8 (100%)	
Difficult ventilation			
Yes	7 (46.7%)	8 (53.3%)	0.188 ^b^
No	53 (64.6%)	29 (35.4%)	

Results are presented as count (%) or mean ± sd; ^a^ *t*-test; ^b^ Pearson Chi-squared test; ^c^ Fisher’s Exact test; ^d^ Chi-squared test for trends.

**Table 5:** Anthropometric parameters of the jaw and neck based on intubation difficulty.

	Easy Intubation (*n* = 60)	Difficult Intubation (*n* = 37)	*p* Value
Anthropometric measurements of the jaw and neck (mm)			
HMDn	32.3 ± 6.6	29.3 ± 6.1	0.025 ^a^
HMDe	45.1 ± 7.7	41.4 ± 7.3	0.021 ^a^
LCD	22.2 ± 2.3	22.9 ± 3.6	0.228 ^a^
TMA	63.7 ± 7.1	61.9 ± 9.1	0.277 ^a^
TMD	43.2 ± 10.2	38.8 ± 7.5	0.024 ^a^
RHTMD	15.0 ± 3.6	16.2 ± 3.3	0.125 ^a^
SMD	66.3 ± 10.4	58.6 ± 8.4	<0.001 ^a^
RHSMD	9.6 ± 1.8	10.6 ± 2.1	0.010 ^a^
NC	232.4 ± 26.8	232.6 ± 29.4	0.967 ^a^
NL	54.9 ± 14	50.1 ± 11.9	0.091 ^a^
Mandibular development			
Normal jaw	51 (71.8%)	20 (28.2%)	0.001 ^b^
Small, hypoplastic jaw	9 (34.6%)	17 (65.4%)	
Mouth opening width (mm)			
>40	25 (80.6%)	6 (19.4%)	0.009 ^d^
20–40	35 (53.0%)	31 (47.0%)	
Neck motility (in degrees)			
Over 120	55 (64.7%)	30 (35.3%)	0.202 ^c^
60–120	5 (41.7%)	7 (58.3%)	

Results are presented as count (%) or mean ± sd; ^a^ *t*-test; ^b^ Pearson Chi-squared test; ^c^ Fisher’s Exact test; ^d^ Chi-squared test for trends.

**Table 6:** Intubation difficulty models.

	Univariable		Multivariable Backward	
	OR (95% CI)	*p* Value	OR (95% CI)	*p* Value
BMI	0.984 (0.970–0.998)	0.028	0.977 (0.957–0.998)	0.031
ASA	3.955 (1.988–7.866)	0.001	18.133 (3.680–89.344)	<0.001
BOV	2.377 (1.417–3.985)	0.001		
CL	4.760 (2.422–9.355)	0.001	3.784 (1.473–9.720)	0.006
HMDn	0.927 (0.866–0.992)	0.028		
HMDe	0.937 (0.885–0.991)	0.024		
TMA	0.971 (0.921–1.024)	0.274	1.224 (1.087–1.377)	0.001
TMD	0.945 (0.898–0.994)	0.029		
RHTMD	1.097 (0.974–1.236)	0.128		
SMD	0.920 (0.877–0.966)	<0.001	0.783 (0.687–0.893)	<0.001
Small hypoplastic jaw	4.817 (1.846–12.57)	0.001		
Mouth opening width 20–40 mm	3.690 (1.339–10.170)	0.012		
Neck motility 60–120	2.567 (0.75–8.789)	0.133		

**Table 7:** Receiver operating characteristic curve values with cut-off values for pre-anesthetic airway assessment methods.

		Area (95% CI)	*p* Value	Sn/Sp	Cut-Off (mm)
Difficult intubation parameters	HMDn	0.650 (0.539–0.761)	**0.013**	0.517/0.838	30.5
	HMDe	0.630 (0.517–0.743)	**0.032**	0.459/0.767	40.5
	LCD	0.478 (0.346–0.610)	0.716	0.432/0.717	20.5
	TMA	0.585 (0.463–0.707)	0.160	0.676/0.500	64.3
	TMD	0.627 (0.515–0.738)	**0.037**	0.892/0.333	46.8
	RHTMD	0.609 (0.496–0.723)	0.071	0.676/0.600	15.3
	SMD	0.714 (0.613–0.816)	**<0.001**	0.757/0.600	64.8
	RHSMD	0.645 (0.532–0.758)	**0.017**	0.703/0.550	9.93
Difficult ventilation parameters	HMDn	0.477 (0.331–0.624)	0.780	0.867/0.305	34.0
	HMDe	0.472 (0.327–0.617)	0.731	0.867/0.305	47.0
	LCD	0.455 (0.308–0.603)	0.583	0.933/0.134	25.5
	TMA	0.676 (0.542–0.811)	**0.030**	0.867/0.488	64.3
	TMD	0.620 (0.484–0.756)	0.141	0.933/0.402	42.0
	RHTMD	0.600 (0.431–0.769)	0.222	0.600/0.707	17.1
	SMD	0.685 (0.546–0.825)	**0.023**	0.800/0.549	63.0
	RHSMD	0.634 (0.469–0.798)	0.101	0.733/0.610	10.42

CI: Confidence interval, Sn: Sensitivity, Sp: Specificity, HMDn: hyomental distance in neutral position, HMDe: hyomental distance in max extension, LCD: lower-lip-to-chin distance, TMA: Tragus-to-mouth angle, TMD: Thyromental distance, RHTMD: Ratio of height to thyromental distance, SMD: Sternomental distance, RHSMD: ratio of height to sternomental distance; significant values are in bold format.

## Data Availability

The data that support the findings of this study are available from the corresponding author upon reasonable request due to ethical restrictions.
